# Euler‐Lagrange computational fluid dynamics for (bio)reactor scale down: An analysis of organism lifelines

**DOI:** 10.1002/elsc.201600061

**Published:** 2016-09-14

**Authors:** Cees Haringa, Wenjun Tang, Amit T. Deshmukh, Jianye Xia, Matthias Reuss, Joseph J. Heijnen, Robert F. Mudde, Henk J. Noorman

**Affiliations:** ^1^ Transport Phenomena SectionDepartment of Chemical EngineeringDelft University of TechnologyDelftThe Netherlands; ^2^ State key laboratory of Bioreactor EngineeringEast China University of Science and Technology (ECUST)ShanghaiPeople's Republic of China; ^3^ DSM Biotechnology CenterDelftThe Netherlands; ^4^ Stuttgart Research Center Systems Biology (SRCSB)University of StuttgartStuttgartGermany; ^5^ Cell Systems EngineeringDepartment of BiotechnologyDelft University of TechnologyDelftThe Netherlands; ^6^ Bio Separation TechnologyDepartment of BiotechnologyDelft University of TechnologyDelftThe Netherlands

**Keywords:** CFD, Euler‐Lagrange, Fermentation, Industrial scale, Scale down

## Abstract

The trajectories, referred to as lifelines, of individual microorganisms in an industrial scale fermentor under substrate limiting conditions were studied using an Euler‐Lagrange computational fluid dynamics approach. The metabolic response to substrate concentration variations along these lifelines provides deep insight in the dynamic environment inside a large‐scale fermentor, from the point of view of the microorganisms themselves. We present a novel methodology to evaluate this metabolic response, based on transitions between metabolic “regimes” that can provide a comprehensive statistical insight in the environmental fluctuations experienced by microorganisms inside an industrial bioreactor. These statistics provide the groundwork for the design of representative scale‐down simulators, mimicking substrate variations experimentally. To focus on the methodology we use an industrial fermentation of *Penicillium chrysogenum* in a simplified representation, dealing with only glucose gradients, single‐phase hydrodynamics, and assuming no limitation in oxygen supply, but reasonably capturing the relevant timescales. Nevertheless, the methodology provides useful insight in the relation between flow and component fluctuation timescales that are expected to hold in physically more thorough simulations. Microorganisms experience substrate fluctuations at timescales of seconds, in the order of magnitude of the global circulation time. Such rapid fluctuations should be replicated in truly industrially representative scale‐down simulators.

**Abbreviations**
CFD: computational fluid dynamics
DO: dissolved oxygen
E: excess (regime)
L: limitation (regime)
S: starvation (regime)
SD: scale down

## 1 Introduction

Nonideal mixing in industrial bioreactors may lead to several large‐scale gradients, for example in substrate concentration, in dissolved oxygen (DO) concentration and in pH. From the point of view of the organisms, these spatial gradients in the reactor translate to temporal variations in their observed environment to which they are continuously subjected 1, and which will influence their metabolism. In order to properly assess the performance and feasibility of industrial bioprocesses upfront, the influence of these variations must be taken into account. This can be done via the use of so‐called “scale‐down (SD) simulators” 2, 3. The design parameters and operating conditions of these simulators are currently often chosen on the basis of intuition or engineering correlations, for example the circulation rate is often based on the vessel mixing time 4, 5, 6, 7, or chosen as a variable 8, 9. Whether the magnitude and frequency of fluctuations observed by organisms based on this assumption are representative is, however, questionable.

A more rational design of SD simulators requires deeper insight in the large‐scale conditions to which organisms are exposed. Unfortunately, industrial equipment is typically poorly accessible for detailed measurements. With state of the art computational fluid dynamics (CFD), it *is* possible to obtain detailed insight in the environment inside the fermentor 10, 11, 12. Of course, such methods involve several assumptions in the modelling of turbulent and multiphase flows and are not perfect in their accuracy, but they provide a significant step forward compared to the information that is currently available experimentally.

Several authors have suggested the use of CFD to tune SD simulators 10, 13, 14, 15, in particular the use of Euler‐Lagrange CFD. In the Euler‐Lagrange method the biomass phase is represented by a set of individual particles, which provides the most straightforward way to study the environmental variations from the perspective of the microorganisms. For each particle, a *condition versus time* series describing the observations of a single microorganism is recorded, referred to as a lifeline, a term coined by Lapin et al. 16. Although the focus here is on the extracellular environment, lifelines for intracellular conditions can similarly be attained 10, 16.

Since the pioneering work of Lapin, who first presented the Euler‐Lagrange methodology 10, 16, only few authors have applied this method, and little attention has been devoted to analysing fermentation simulations from the unique microbial perspective offered by the approach. Lapin et al. and Delvigne et al. 13 showed lifeline plots, but did not quantify fluctuation frequencies. Some initial quantification of substrate concentration variations, considering both frequency and magnitude, has been conducted by McClure et al. 17. Still, to our knowledge, no extensive statistical analysis of CFD‐based lifelines has been published to date. Such substrate concentration fluctuation statistics are of great value for the design of representative SD simulators as they provide deeper insight in what conditions organisms experience in industrial scale fermenters and can therefore provide a basis of design for industrially representative SD simulations. The major challenge in this respect is to transform the large amount of simulation data to a manageable set of statistics. This paper aims at developing a methodology to address this issue. As such, we do not claim that the CFD results shown in this paper are a complete representation of the fermentation environment. For instance, we ignore the presence of a bubbly flow and the associated oxygen transfer, assuming sufficient oxygen is present. Furthermore, the complex, transient rheology of the broth is omitted. These simplifications do, however, not affect the methodology we develop; to illustrate what organisms may encounter in a large‐scale fermentor it suffices to roughly capture the relevant timescales of mixing and reaction. In this paper, we present a methodology to collect statistics insight in environmental (substrate) variations observed by microorganisms that may serve as a basis of design of SD simulators.

## 2 Materials and methods

We applied an Euler‐Lagrange CFD approach to study the extracellular environment in an industrial scale fermentor from the microbial viewpoint, focussing on the extracellular glucose concentration $C_{s}\;$(mol/kg_broth_). All other conditions are assumed constant or noninfluential in this study. Extracellular variations in $C_{s}\;$lead to variations in the biomass specific substrate uptake rate $q_{s}$ (mol*_s_*/*C*mol*_x_*/hr) for each individual organism. Since we are interested in the response of the microorganism, the lifelines are expressed in terms of $q_{s}$ versus *t*.

Our study is based on a 54 m^3^ *Penicillium chrysogenum* fermentation in a reactor formerly operated by DSM. To predict the dynamic response of $q_{p}$, the biomass specific penicillin production rate, we apply the dynamic gene regulation model developed by Douma et al. 18 for strain *DS12975*. Originally, this model was developed for slow dynamics in an otherwise ideally stirred fermentor, rather than the rapid variations induced by imperfect mixing. Whether or not the predictions of the Douma model hold for rapid extracellular dynamics is an issue not further addressed in this work. We have refrained from the use of more complex kinetic models 10 in order to focus on lifeline analysis.

### 2.1 Biomass specific kinetics

The substrate uptake rate of *P. chrysogenum* is modelled using a hyperbolic relation, Eq. (1)

(1) $$q_{s}\;=\;q_{s,\max}\cdot \left(\frac{C_{s}}{K_{s}+\;C_{s}}\right)\;\left(mol_{s}/Cmol_{x}/s\right)$$

De Jonge et al. 5 determined values of $q_{s,\max}=\;12.47\cdot \;10^{-6}\;$mol*_s_*/*C*mol*_x_*/s and $K_{s}\;=\;7.8\cdot 10^{-6}$ mol*_s_*/kg for *DS12975*. The *q*_p_dynamics follow from Eq. (2) 18. Growth, production, and maintenance are linked to *q*_s_ via the Herbert–Pirt, Eq. (3).

(2) dqpdt=β·max0,μ1+CsKp2−K dE +μqp

(3) qs=μY sx +qpY sp +ms

In Eq. (3), the term qp/Y sp is small and can be safely neglected to make μ a function of *C*_s_ only. Some modifications to the model parameters and equations had to be made in order to reconcile the work of Douma and De Jonge, and to prevent nonphysical responses at very low *q*_s_. These alterations are detailed in Section A of the Supporting Information.

#### 2.1.1 Oxygen dynamics

We currently assume sufficient oxygen is supplied and do not consider DO gradients. It is known for several *P. chrysogenum* strains that *q*_p_ is affected below DO ≈ 0.08 mol/m3 and production may be lost below 0.026 mol/m^3^. The reversibility of this loss is disputed 4, 19, 20. How low DO affects *q*_s_ is not well known; Henriksen et al. observed no significant change in μ and the residual glucose concentration for low DO 20 while McIntyre et al. did observe a significant reduction in *C*_x_ under complete starvation 21 (they did not report on residual glucose concentrations). We have no data available regarding DO levels for the 54 m^3^ fermentor. However, in a comparable penicillin process in a 150 m^3^ vessel, the registered DO in the top was approximately a factor 2 lower than in the bottom, with a minimum of 0.05 mol/m^3^ toward the end of the fermentation. Although the lowest values were below the level at which *q*_s_ may be affected and we cannot comment on the possible formation of local depletion pockets, these values do not indicate very serious oxygen limitation in the 150 m^3^. Hence, we do not expect serious limitations in the smaller vessel either.

#### 2.1.2 Metabolic regimes

Both *q*_s_ and μ are nonlinear functions of $C_{s}$, saturating for $C_{s}\to \infty$. For $C_{s}>19K_{s}$, $q_{s}>0.95q_{s,\max}\;$and both *q*_s_ and μ become largely insensitive to *C*_s_ variations. We regard the domain $C_{s}>19K_{s}$ to be a single “metabolic regime” (a domain in the *C*_s_—space characterized by a certain consistent metabolic response—in this case insensitivity to variations), referred to as the (substrate) excess regime (E). Practically, *q*_s_ can be assumed independent of the extracellular *C*_s_, for organisms exposed to excess conditions. In the domain $C_{s}<K_{s}/19,$ *q*_s_ is a linear function of *C*_s_ with a magnitude $q_{s}<0.05q_{s,\max\;}$. The low absolute magnitude of *q*_s_ in this regime means fluctuations can safely be neglected (We further discuss this assumption in Section A of the Supporting Information). We refer to this low *q*_s_ regime as the starvation regime (S). The domain between excess and starvation is classified as the limitation regime (L); here, *C*_s_fluctuations do lead to nonnegligible variations in *q*_s_. The above considerations lead to the following distinction:

- E: ($q_{s}>0.95q_{s,\max}$).
- L: ($0.05q_{s,\max}<q_{s}<0.95q_{s,\max}$).
- S: ($q_{s}<0.05q_{s,\max}$).

This categorization forms the basis of our lifeline analysis. We will consider two indicators: The exposure time to each of these regimes, and the magnitude of fluctuations within the L, where *q*_s_ *is* sensitive to *C*_s_ variations.

### 2.2 Simulation setup

We considered a 54 m^3^ fermentor (height H=7.7 m, diameter T=3.0 m) with two Rushton turbines (8‐blade bottom, 6‐blade top, diameter D=1.3m) operating at $N_{s}=\;1.63$ s^−1^ and four baffles of width T/10. Substrate solution was fed at the fermentor top with a rate of F=0.37 mol*_s_*/s, the broth density was ρ broth =1000 kg/m^3^ with biomass concentration $C_{x}=\;1.96$ *C*mol*_x_*/kg (55 g/kg_broth_ dry matter). In the industrial study of Goldrick et al. 22 the broth weight and Fwere constant after 80 h^−1^ with $C_{x}$ approximately constant after 150 h^−1^. The process at hand had a shorter filling stage (≈1day) and higher *C*_x_, but qualitatively similar dynamics apply. As such, our simulation choices (constant $H,\;F,\;C_{x}$) represent the mid/late fermentation stage. The feed rate *F* was set to optimize *q*_p_ under the assumption of ideal mixing conditions (Section 3.1) to facilitate comparison of the predicted *q*_p_ under ideal and non‐ideal conditions. Half of the tank was modelled by imposing periodic boundary conditions. This does impose 2 adjacent glucose feed locations rather than a single feed. Due to compartment formation (zoning) by Rushton turbines the glucose gradient is mostly axial in this case 10, 23, 24, 25 and the duplicate feed points do not result in a significantly different concentration field.

Mixing experiments have been performed by DSM by supplying concentrated H_2_SO_4_ via the top and measuring pH response with a probe mounted near the bottom. These experiments have been performed aerated and nonaerated water and broth (a strain similar to *DS12975*) as working fluids. The circulation behaviour was quantified by measuring the time lag between feeding and detection, this lag time is half the representative circulation time for a single loop in the vessel and is given in Table 1 for several situations. The circulation time is related to the 95% mixing time (τ_95_) via the rule of thumb τ95≈4τ circ  26. No full tracer response curves were available for the industrial vessel, hence we could not validate $\tau_{95\;}$and mixing dynamics.

**Table 1:** Experimental values for the circulation time under different flow conditions

Fluid	U sup , gas [m/s]	τ circ [s]
Water	0	19.26
Water	0.05	42.8
Broth	0	77
Broth	0.05	25.6

*U*_sup, g_, superficial gas velocity. Numerically, τ circ =18.2s for the nonaerated water case (see Section B of the Supporting Information for details).

Penicillin broth is viscous and shear thinning, and high aeration rates are employed. Modelling of these complex fluid dynamics has been attempted with varying success 24, 27, 28. For example, the mixing time was strongly overpredicted by Moilanen et al 28, being very sensitive to stagnant zones and hence rheology–turbulence interaction. Furthermore, bubble size population balances are typically developed and validated for air‐water flows and their applicability to broths is poorly tested. Further developments regarding the simulation of transitional, aerated, non‐Newtonian flows are required for the reliable simulation of viscous fermentations. As we wish to focus on the analysis of organism lifelines rather than the physics of bioreactors here, we opted to simplify the physics in our simulation by modelling a single‐phase water situation.

This assumption is further justified by considering that the circulation time under fermentation conditions is reasonably close to that in nonaerated water (Table 1). Van ’t Riet and van der Lans 29 argue that the mixing time for a gas flow number $0.07<Q_{g}/ND^{3}<0.2$ is approximately equal to that under nonaerated conditions. Furthermore, aeration influences the spatial mixing behaviour due to a change in the dominant transport mechanism 26, 30. The difference in τ_circ_ and flow pattern will affect details in the glucose gradient and residence time distributions in Section 3.6.2 but as the difference between τ_circ_ and the reaction timescale (Section 3.2) is large in both cases, the observations are expected to hold at least qualitatively. The applied simplifications do not compromise the goal of our current work; to show how the data from Euler–Lagrange CFD can be analyzed in order to study to which extracellular variations microorganisms are subjected.

### 2.3 Hydrodynamics

We applied the well‐validated RANS approach used by several earlier studies: The k−ε model for turbulence modelling in combination with the multiple‐reference frame for impeller rotation 31, 32. Substrate transport is modelled by Eq. (4) as follows:

(4) $$\frac{\partial C_{s}}{\partial t}\;+\;\nabla \cdot \;\left(uC_{s}\right)=\;-\nabla \cdot (-\left(\left(D+\frac{\nu_{t}}{Sc_{t}}\right)\nabla \;C_{s}\right)\;+\;S_{s}$$

The turbulent Schmidt number $Sc_{t}$ was set to 0.2to achieve agreement between the computed and experimental mixing time, as noted by 24, 25, 33. In all other aspects, we followed the recommendations by Gunyol and Mudde 31 for flow modelling.$\;\;N_{p}=\;175\;000$ particles were tracked for 1700 s flow time. These numbers were chosen to ensure a rapid convergence of the statistics. Computationally, the particles were treated as massless tracers, instantly adapting to the convective flow velocity 16. Turbulent motions were superimposed on the convective velocity using the discrete random walk model. For each particle, *q*_s_ was stored every Δt=0.03 s, yielding the organism lifelines. Further details on the CFD settings and particle tracking method can be found in Section B of the Supporting Information.

## 3 Results and discussion

We begin with a reactor‐level analysis, to establish a background on which to project observations from the microbial perspective.

### 3.1 The ideal mixing reference

The steady‐state ideal mixing assumption, yielding Eq. (5), gives a residual glucose concentration $C_{s,\mathrm{res}\;}=\;3.06\cdot 10^{-6}$ mol/kg.

(5) F=Vρ·Cx·qs, max Cs, res Ks+Cs, res .

From Eqs. (1)–(3) steady values of $q_{s}=\;3.51\cdot 10^{-6}$ mol*_s_*/*C*mol*_x_*/s, μ=0.045 h^−1^ and $q_{p,\mathrm{ideal}}=\;5.2\cdot \;10^{-4}$ mol*_p_*/*C*mol*_x_*/h are obtained. As noted earlier, we assumed $C_{x},\;F,\;H$ in these calculations. Whether *C*_x_ is truly constant despite a positive μ depends on dilution effects and on cell death kinetics, which we do not explicitly consider here. As a constant *C*_x_facilitates a more direct comparison of $q_{p}\;$under homogeneous and heterogeneous substrate conditions by making $C_{s\;}$the only variable, the constant *C*_x_ assumption is made mainly for practical reasons.

### 3.2 Reactor level simulation results

**Figure 1:**
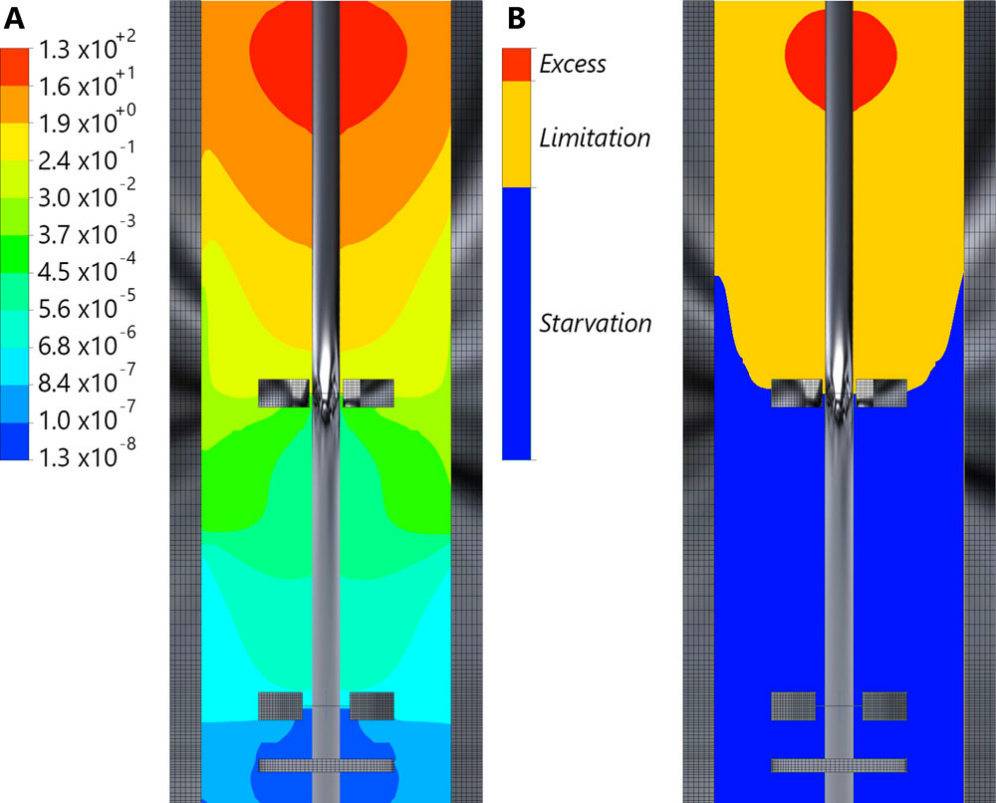
Gradients in the Eulerian simulation of a 54 m^3^ fermentor; (A) log‐contours of $\frac{C_{s}}{K_{s}}$. (B) Volumetric distribution (linear scale) of the specific substrate uptake regimes. Red: E, with $\mu >0.95q_{s,\max}$; Blue: S $\mu <0.05q_{s,\max}$; Yellow: L.

**Eulerian perspective**: ***C***_s_, ***q***_s_, **and** ***μ***: The representative timescale for substrate consumption reads $\tau_{r,s}=\frac{C_{s}}{C_{x}\cdot q_{s}}\;=\;0.444$ s for the ideal mixing case; significantly shorter than τ circ ≈19 s. The consequence is a substrate concentration gradient spanning orders of magnitude (Fig. 1A). Due to rapid consumption in the top, organisms are exposed to starvation conditions in a significant region of the vessel. This is reflected in the spatial regime distribution, Fig. 1B.

Because diverging jets of a Rushton impeller act as a barrier against axial mixing 34, a *C*_s_ jump is expected in the impeller plane. Consequentially, the limitation‐starvation boundary coincides with the top impeller here; a twofold increase in *F* is required for the boundary to cross this barrier. Unfortunately, we lack experimental data to verify such details of the substrate gradient. Using a similar setup, Gunyol et al. showed a good prediction of the substrate gradient in a 22 m^3^ *S. cerevisiae* fermentation 25, giving confidence in the approach. The strong gradient observed here is mainly a consequence of the low *K*_s_ value. Even when *q*_s_ is significantly lower in practice, for example due to oxygen limitation, a strong gradient is still expected: very large changes in *q*_s_ or *C*_s_ are required to yieldτ circ ≈τ rxn .

Both *q*_s_ and μ, being instantaneous functions of *C*_s_, are widely distributed in the reactor. Still, the average $\bar{q_{s}}\;$and $\bar{\mu }$ are equal to the value under ideal mixing conditions as a simple consequence of the overall substrate uptake balance. In contrast, the mean substrate concentration $\bar{C_{s,c}}=\;34.4\cdot 10^{-6}$ mol/kg, over a factor 10 higher than the ideal mixing value. This results from the saturated *q*_s_ in the top as a consequence of poor mixing, with high *C*_s_ regions strongly impacting $\bar{C_{s,c}}$. Close to the feed, $C_{s}\;$and $q_{s}\;$are essentially decoupled allowing for these high $C_{s}$ regions to exist.

### 3.3 $q_{p}$ under fluctuating conditions

For illustrative purposes the possible impact of fluctuations we determine *q*_p_ under fluctuation conditions. The resolved flowtime of 1700 s is, however, much shorter than the adaptation time of *q*_p_, τ qp =1K dE +|μ|≈20 h. Assuming statistical similarity, we combined the 175 000 resolved lifelines to form 1400 lifelines spanning t≈59 h. Assuming constant $C_{x},\;F,\;H$ based on 22, a steady state of $q_{p}=\;0.77\cdot \;10^{-4}$ mol_p_/*C*mol_x_/h is reached under nonideal conditions; an 85% reduction compared to the ideal mixing estimation. Owing to the large difference between τ_circ_ and the τ_qp_ no notable heterogeneity in $q_{p}\;$within the population was observed. Using a steady$\;\;\bar{C_{s,c}}=\;34.4\cdot \;10^{-6}$ mol/kg yields $q_{p}=\;0.29\cdot \;10^{-4}\;$mol_p_/*C*mol_x_/h, clearly showing that the Eulerian mean is not a representative parameter.

### 3.4 Validation of the Lagrangian point of view

The mean substrate concentration observed from population point of view,$\;\;\bar{C_{s,P}}=\;32.9\cdot \;10^{-6}\;$mol/kg agrees well with the Eulerian $\bar{C_{s,c}}=\;34.4\cdot \;10^{-6}\;$mol/kg indicating that the particles are properly, spatially homogeneously, distributed. The small difference is attributed to weaknesses in the particle turbulence model (see Section B of the Supporting Information). There is a similar level of agreement in the spatial regime distribution (Table 2). Turbulence filtering of the particle tracks, required for a tractable lifeline analysis (Section 3.6.1), affects the regime distribution slightly but with a maximum 5% difference from the Eulerian distribution the margin is acceptable.

**Table 2:** Volumetric distribution of the metabolic regimes in the fermentor

Case	% Starvation	% Limitation	% Excess
Eulerian	57.0	36.2	6.8
Lagrangian, raw	57.4	35.9	6.7
Lagrangian, filtered	58.2	34.7	7.1

The turbulence‐filtered Lagrangian case is detailed in Section 3.6.1.

### 3.5 Dynamics of individual lifelines: Fourier analysis

The suggestion to decompose organism lifelines using Fourier analysis has been made in several unpublished talks by Reuss. There are reasons to be sceptical toward the applicability of Fourier analysis for this particular case as the circulation times of particles inside a stirred tank are widely distributed 14, 35 and no dominant circulation frequencies are expected.

**Figure 2:**
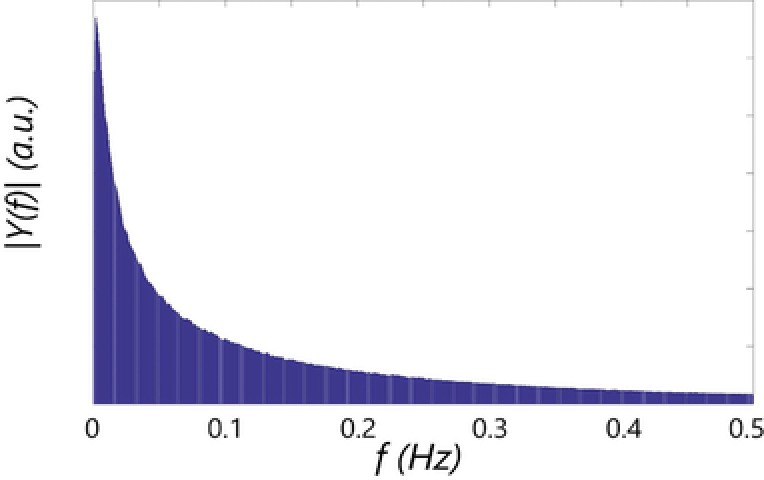
Frequency spectrum of glucose variations, summed for 175 000 particles.

We took the Fourier transform of each individual *q*_s_‐series, after subtracting the series mean *q*_s_ and multiplication with a *Blackman* window function 36. The per‐track frequency spectra were summed, yielding Fig. 2. As expected, no frequencies stand out due to the wide circulation time distribution (Section 3.6.2), and no more direct insight is gained in the frequency domain. A specific issue is that the applied approach does not discriminate between fluctuation amplitudes; all fluctuations—regardless of their amplitude—are summed in the composite spectrum. There may be ways to overcome this, but it is unlikely to yield a simpler or clearer picture than a conditional analysis in the time‐domain. We hence have chosen to discard the Fourier analysis in favour of a time‐domain approach, where we discriminate variations based on the metabolic regimes described in Section 2.1.2.

### 3.6 Dynamics of the individual lifelines: Metabolic regime analysis

#### 3.6.1 Lifeline analysis methodology

Our analysis of microorganism lifelines draws heavily from the techniques used in circulation time determination 14, 35, which are based on consecutive crossings of a region in space. Since microorganisms have no notion of their physical location, we use $q_{s}-\mathrm{space}\;$as a more relevant space for the determination of residence time/circulation time distributions regarding variations in *q*_s_. Using the regimes of Section 2.1.2 as a basis, the regime residence time is defined as the time spent by a microorganism inside a certain regime, between two consecutive crossings of the regime boundaries. A second central concept in our analysis is transition patterns: These are determined by the nature of the two consecutive crossings.

**Figure 3:**
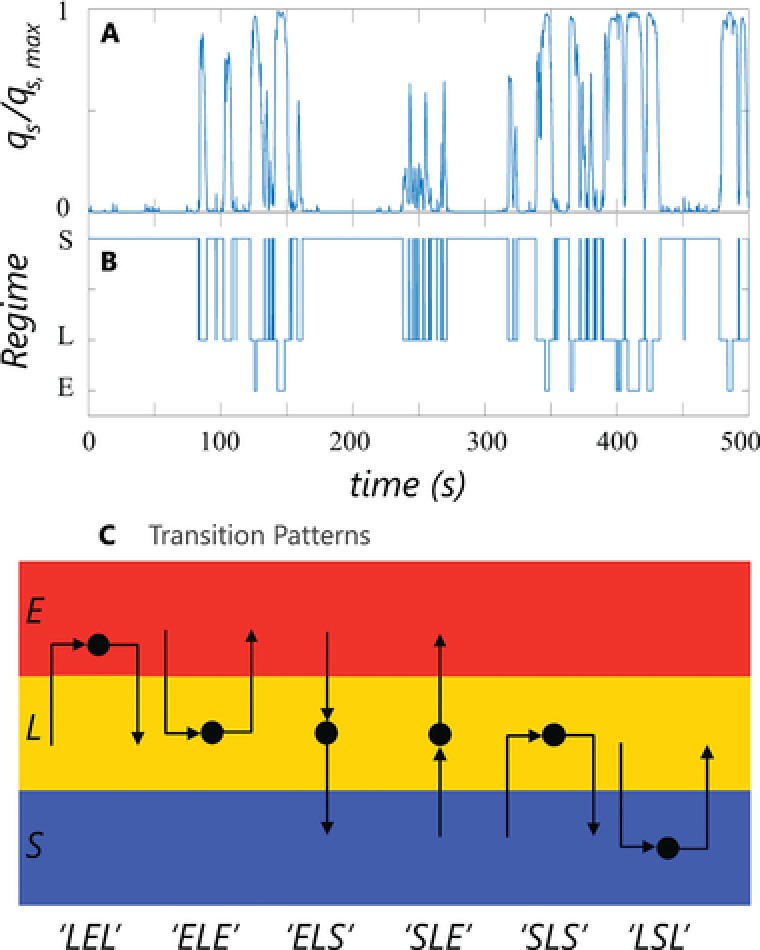
Processing steps to determine residence times and transitions. (A) Original *q*_s_ signal. (B) Regime‐signal based on the regimes of Section 2.1.2. (C) Graphical depiction of the 6 possible transition patterns. Each transition is coded by the regime of origin (first), the regime the organism resides in (middle), and the regime of destination (last). E: excess; L: limitation; S: starvation.

After preprocessing with turbulence filters (explained later in this section), the $\frac{q_{s}}{q_{s,\max}}\;\left(t\right)$ series as shown in Fig. 3A, is converted to regime series, Fig. 3B. From this series, it is straightforward to determine the regime residence time, the time between two boundary crossings, and the transition pattern: the nature of the two successive crossings. We distinguish between six of such transition patterns as follows:

- LEL: From limitation, in excess, to limitation.
- ELE: From excess, in limitation, to excess.
- ELS: From excess, in limitation, to starvation.
- SLE: From starvation, in limitation, to excess.
- SLS: From starvation, in limitation, to starvation.
- LSL: From limitation, in starvation, to limitation.

Here, the middle letter indicates the regime in which the particle is residing, the first and last their origin and fate, respectively. For example, a 6‐s “ELE” event means that a particle originates from excess, spends 6 s in limitation, and then returns to excess. Graphically, the patterns are depicted in Fig. 3C. Due to the physical distance, no direct crossings between excess and starvation (SES or ESE) have been observed. Discriminating between the different transition patterns provides insight in how microorganisms move between regimes and how the regime‐residence time is linked to their trajectory.

Turbulence filters are applied on the $\frac{q_{s}}{q_{s,\max}}\;\left(t\right)\;$signal, consisting of a moving‐average smoothing and low‐amplitude filtering step. These are applied to remove rapid, low amplitude oscillations$\;q_{s}$ caused by turbulent movements. These skew the residence time statistics by introducing sharp peaks at short timescales, probably with limited metabolic impact, and which will be difficult to reproduce in SD simulators explicitly. Details of the filtering procedure and a brief analysis of turbulent $q_{s}$ oscillations are presented in Section C of the Supporting Information. To summarize, our regime analysis consists of four steps as follows:

- Nondimensionalise *q*_s_ with *q*_s, max_.
- Turbulence filtering: smoothing/amplitude filter.
- Conversion to regime vector.
- Determination of transitions and residence times.

#### 3.6.2 Per‐regime residence time distributions

**Figure 4:**
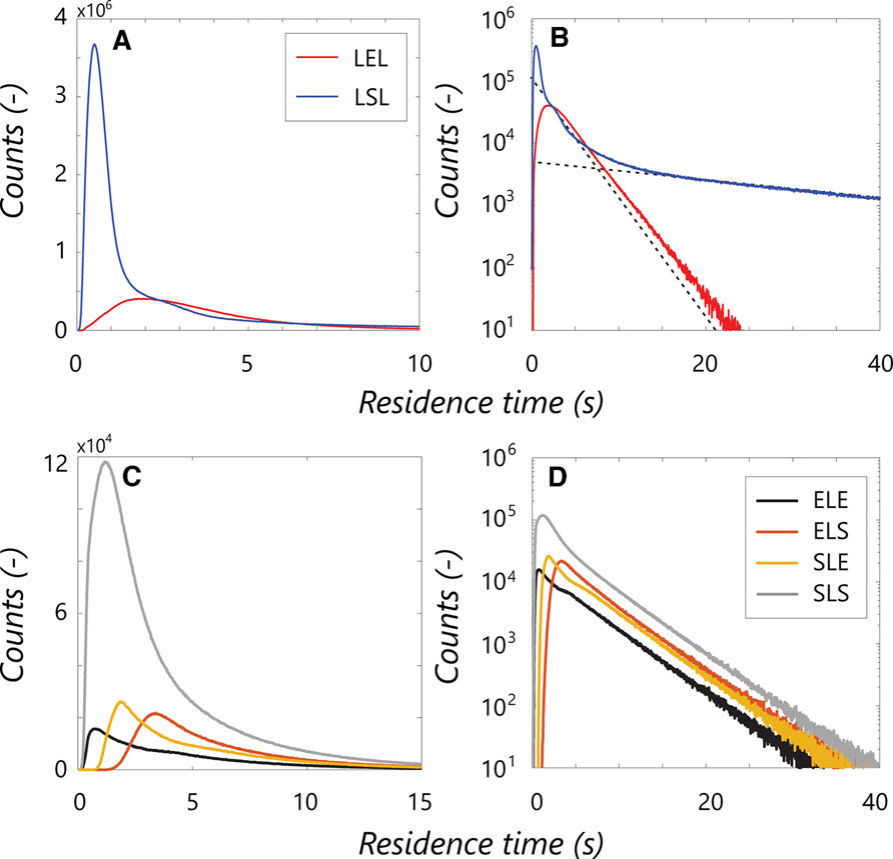
(A, B) Nonnormalized residence time distributions for the excess (*LEL*) and starvation (*LSL*) regime. Dashed lines: the two dominant circulation modes for the ‘*LSL*’ distribution. (A) Short timescales, (B) long times. (C, D) Nonnormalized residence time distributions for the four different transitions patterns through the L. (C) Short timescales, (D) long times. The mean residence times are $\bar{\tau_{r,\mathrm{LEL}}}=\;3.65,\;\;\bar{\tau_{r,\mathrm{LSL}}}=\;9.37,\;\;\bar{\tau_{r,\mathrm{ELE}}}=\;4.67,\;\;\bar{\tau_{r,\mathrm{ELS}}}=\;6.45,\;\;\bar{\tau_{r,\mathrm{SLE}}}=\;5.39,\bar{\tau_{r,\mathrm{SLS}}}=\;3.77$.

The key figure in the regime residence time discussion is Fig. 4, showing the excess and starvation residence time distributions (Fig. 4A and B) and distribution for the four different limitation transitions (Fig. 4C and D). For a more direct comparison between the curves, the distributions are nonnormalized. The distribution for the E, “LEL” (Fig. 4A and B), is rather straightforward: Initially there is a gradual increase in counts, associated with trajectories directly crossing the “LEL” regime, of varying duration. The constant slope in the *log‐lin* plot (Fig. 4B) is indicative of an exponential decay for t>5 s representing circulation *inside* the excess region, with the probability of leaving the regime at a certain time becoming independent of the incoming trajectory.

**Figure 5:**
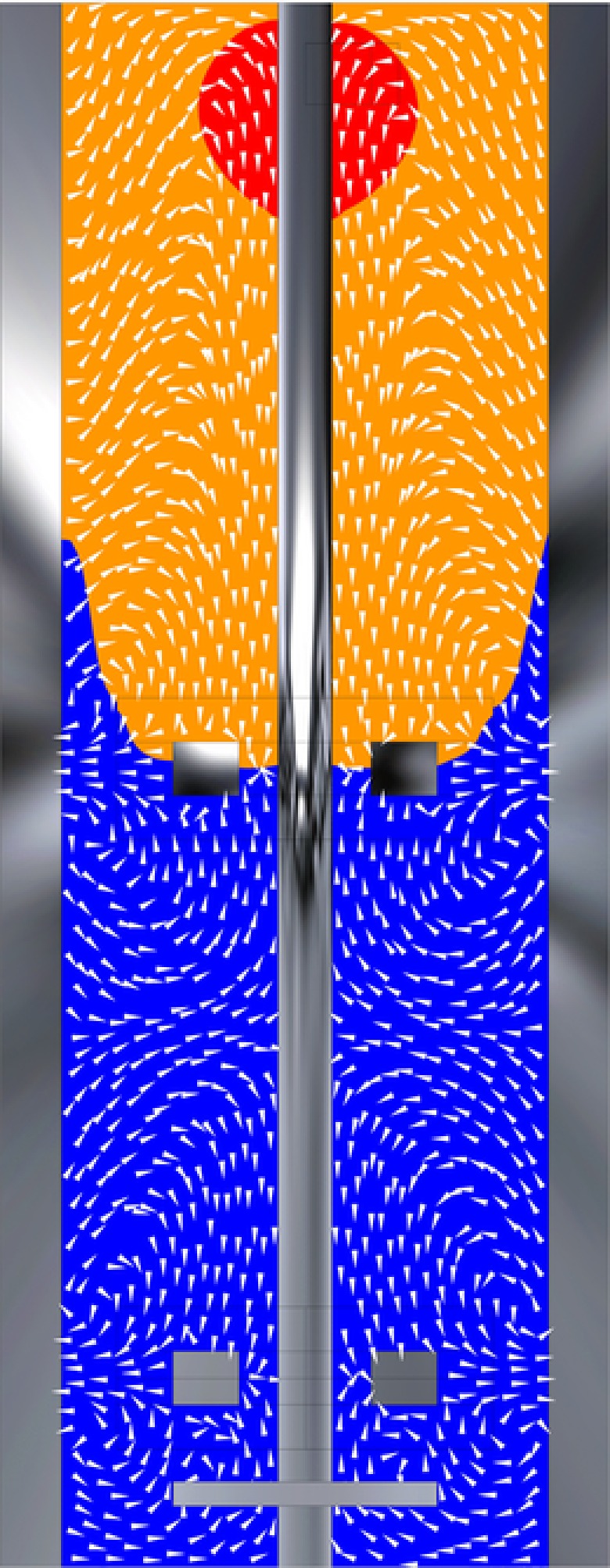
Metabolic regimes (equal to Fig. 1) with superimposed velocity vectors. The vectors only show flow direction, not magnitude.

As shown in the *log‐lin* plots, all six distributions show similar exponential decay behaviour at long timescales. The long‐time behaviour of the starvation distribution, “LSL,” is particularly interesting. After an initial peak, in Fig. 4B two slopes are distinguished; for t res <10 s the distribution exhibits decay at a slope roughly equal to that of the “LEL” distribution, gradually changing to a much weaker slope for t res >20s, indicating two circulation modes with different representative timescales. These modes can be understood from the regime distribution combined with the flow field (Fig. 5). Particles move from limitation to starvation at the top impeller, where three situations can occur as follows: (i) The particle follows the upward‐circulation loop and is exposed to starvation conditions briefly before reentering to limitation. (ii) The particle follows the downward circulation loop. Upon returning to the impeller it can move back to limitation, or recirculate in starvation. (iii) The particle crosses to the bottom impeller region and recirculates under starvation conditions for a long time. The recirculation behaviour at t res <10s is associated with option ii, the behaviour at t res >20s with option iii, with overlap in between. The sharp peak at t res ≈0.7 s in Fig. 4A is associated with option i; all particles in the upward loop of the top impeller outflow are very briefly exposed to starvation conditions (Fig. 5). Similarly, trajectories originating from, and moving back to starvation via the downward circulation loop of the top impeller, result in an “SLS” peak at t res ≈1 s (Fig. 4C). Considering the four different limitation residence time distributions, details such as the abovementioned “SLS” peak yield differences at short times (Fig. 4C), but the four distributions shown an equal‐sloped decay after approximately 5 s. This is again consistent with the notion that particles end up in origin‐independent recirculation behaviour if not crossing the limitation zone directly.

From summation of the counts it follows that 39% of the trajectories starting in excess move back to excess (the “ELE” transition). Similarly, 80% of trajectories starting in starvation are of the “SLS” type. Clearly, on many occasions organisms will repeatedly oscillate between limitation and starvation conditions before being exposed to excess conditions. In contrast, prolonged oscillation between excess and limitation is less likely. Evidently, such sequential regime transitions should be reflected in an experimental SD setup.

#### 3.6.3 *q*_s_ dynamics in the Limitation regime

When exposed to limitation conditions, the magnitude of *q*_s_ influences the metabolism of a microorganism. We study the duration and magnitude of these intralimitation variations by considering (i) the time between registrations of $\frac{q_{s}}{q_{s,\max\;}}=\;0.5,$ referred to as the *arc time* (τ_arc_) and (ii) the relation between τ_arc_ and the observed extreme value $\frac{q_{s}}{q_{s,\max\;}}$ over the arc trajectory.

##### 3.6.3.1 Arc‐time distribution

The arc time provides a timescale for the duration of fluctuations *within* the L. We distinguish between arcs moving up from the baseline $\frac{q_{s}}{q_{s,\max\;}}=\;0.5$ with timescale τ arc ,+, and moving down with timescale τ arc ,−. Note that the distribution in τ_arc_ presented here is only valid when using $\frac{q_{s}}{q_{s,\max\;}}=\;0.5$ as a baseline; a different baseline may be chosen, as long as it is consistent between the simulation and SD experiment.

**Figure 6:**
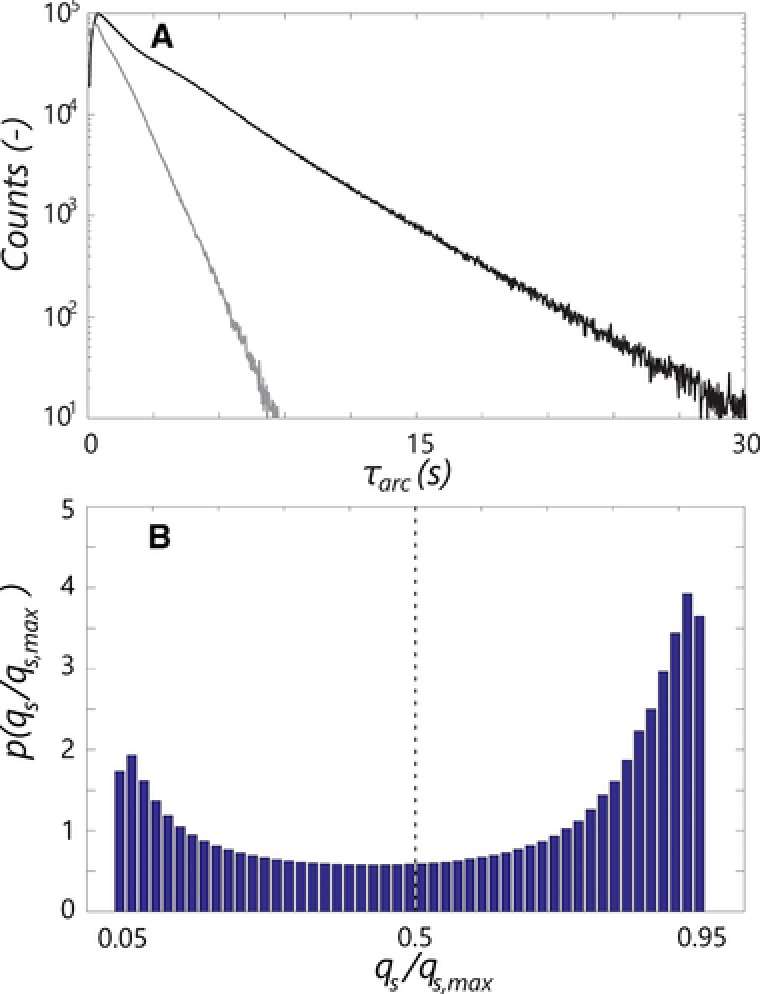
(A) Distribution of τ arc ,+ (black) and τ arc ,− (gray), the duration of upward and downward fluctuations in *q*_s,_ compared to $q_{s}/q_{s,\max}=\;0.5$. Only trajectories completely within the L are counted. (B) Normalized histogram of $q_{s}/q_{s,\max}$ in the limitation domain of the reactor.

In Fig. 6A, the distributions for τ arc ,+ and τ arc ,− are shown, again nonnormalized. The average arc times τ arc ,+¯=3.14s and τ arc ,−¯=1.11 s, demonstrating an asymmetry in the up‐ and downward trajectories. These numbers are understood from the $\frac{q_{s}}{q_{s,\max\;}}$ histogram (Fig. 6B), showing that in the majority of the limitation region, $q_{s}>0.5q_{s,max}$. In view of brevity, we will not further delve into this distribution. Of all trajectories, 73% move upward. This is in part due to the asymmetric *q*_s_ distribution mentioned above, and in part due to the action of the top impeller. Most particles moving down from $\frac{q_{s}}{q_{s,\max\;}}=\;0.5$ are drawn into starvation, leading to few downward trajectories fully within the limitation region.

##### 3.6.3.2 Magnitude of ***q***_s_ variations

**Figure 7:**
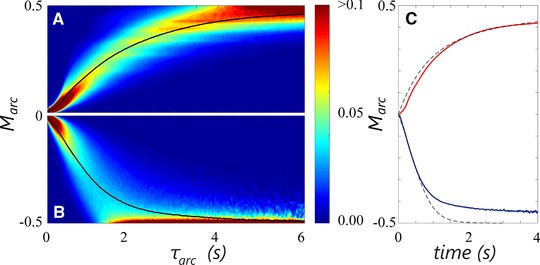
(A) Evolution of *M*_arc_, the maximum $q_{s}/q_{s,\max}$ observed during a fluctuation, as a function of fluctuation duration τ_arc_ for upward fluctuations with respect to $q_{s}/q_{s,\max}=\;0.5$, (B) Same as A, for downward fluctuations. The color bar gives the bin fraction of *M*_arc_ at time τ_arc_. Bin fractions sum to 1 for each time instance. Solid lines: M arc ¯ versus τ_arc_. (C) Comparison between the rate‐of‐change in $q_{s}$ in the industrial simulation, and the rate of change achievable by consumption alone. Red line: M arc ,+¯ vs. $0.6\cdot \tau_{arc,+}$. Blue line: M arc ,−¯ vs. 0.5·τ arc ,−. The factors 0.6 and 0.5 arise from the average arc shape. Dashed lines: Time required for a given change in *q*_s_ by substrate consumption alone, at $C_{x}=\;1.96$ *C*mol/kg.

The magnitude *M*_arc_ is defined as the maximum observed value of $\frac{q_{s}}{q_{s,\max\;}}$ along an arc trajectory, minus the baseline: M arc =maxqsqs, max  arc −0.5. Figure 7 shows the probability distribution of *M*_arc_ as a function of τ_arc_ for upward (top) and downward (bottom) trajectories. The colour scale represents the fraction of counts in each bin, for each τ_arc_, and the mean M arc ¯ versus τ_arc_ is superimposed by the solid lines. Despite considerable spread around the mean, there is clear a connection between M arc ¯ versus τ_arc_ that can be exploited for SD purposes. The continuous nature of the M arc ¯ versus τ arc relation is consistent with the notion that *q*_s_ is strongly heterogeneous within the top circulation loop, where limitation oscillations take place.

To determine the temporal symmetry of the individual arcs we record at which time t max τ arc  the value of *M*_arc_ is registered. The arcs are (on average) symmetric if t max τ arc ¯=0.5. For downward trajectories this is indeed the case. In contrast, upward trajectories give t max τ arc ,+¯=0.4; on average, 40%of the duration is used to change from $\frac{q_{s}}{q_{s,\max\;}}=\;0.5$ to $\max\left(\frac{q_{s}}{q_{s,\max\;}}\right)$ and the remaining 60%to move back to $\frac{q_{s}}{q_{s,\max\;}}=\;0.5$.

In nonideal reactors, both consumption by the microorganisms, and dilution by counter‐current mixing of a substrate‐rich and substrate‐lean stream contribute to the *q*_s_ dynamics observed by organisms. In (ideal plug flow/batch) SD simulators only consumption contributes to the decrease of *q*_s_. Hence, it is interesting to compare how much $\frac{q_{s}}{q_{s,\max\;}}$ decreases in a given time *t* in the simulations, and how much it *can* decrease by consumption alone (for $C_{x}=\;1.96$ *C*mol*_x_*/kg).

Due to the arc shape, the time *t* available to change from maxqsqs, max  arc  to $\frac{q_{s}}{q_{s,\max\;}}=\;0.5$ is 0.6τ arc ,+ for upward arcs and $0.5\tau_{arc,-}$ for downward arcs. These timescales are compared with the time required to make the same change by consumption alone in Fig. 7C where the solid lines are the simulation timescales, the dashed lines the consumption timescales. The agreement between the curves is striking; the rate of change in $q_{s}$ in industrial fermentors seems to closely follow the maximum rate of change that *can* be achieved by consumption alone. Naturally, the agreement breaks down near the regime boundaries, considering only trajectories *within* the limitation region. The significance of the above observation is further discussed in the Section 3.7.

### 3.7 Design of scale down simulators: Outlook

We have provided a comprehensive statistical analysis of the magnitude and duration of variations in simulated extracellular substrate concentrations in industrial bioreactors. The next challenge is to translate these statistics to design parameters for SD simulators. These are typically operated by imposing feed variations in a single vessel 5, or by using multiple compartments with exchange loops 2, 26. There are 5 degrees of freedom for SD simulator design and operation 26, whose value can be determined based on the statistical distributions from our analysis.

In our simulations extracellular conditions fluctuate at timescales of seconds. However, current SD simulators typically employ fluctuation timescales of 100 − 500 s 2, 4, 5, 7, 9 based on$\;\tau_{95}$ 6. We consider τ_circ_ to be more representative for extracellular substrate variations between the extreme values, with smaller fluctuations occurring at even shorter timescales.

An important question is whether the current generation of SD simulators can replicate variations on such short timescales. When the generally applied assumption of ideal mixing (with varying feed) or ideal plug‐flow holds, the rate of change in the extracellular substrate environment is limited by consumption to $q_{s}\cdot C_{x}$. For substrate variations *within* the L, the observed rate of change in our simulation closely follows the maximum rate of change achieved by consumption alone (Section 3.6.3).

Furthermore, the time required to change from $\frac{q_{s}}{q_{s,\max\;}}=\;0.95$ to $\frac{q_{s}}{q_{s,\max\;}}=\;0.05$ by consumption alone is 8.13 s, while the mean residence time for this “ELS” transition reads τ res , ELS ¯=6.45 s. In nonideal systems, the local rate of change observed by an organism locally is the sum of consumption and mixing, which may locally exceed the rate of change by consumption alone. This dilution effect results in an average “ELS” transition that is *faster* than an “ELS” transition by consumption alone. This has a far‐reaching consequence: to mimic τ res , ELS ¯ in an ideal SD simulator, it must operate at a *C*_x_ *higher* than the industrial fermentation it replicates. This is a direct consequence of the kinetics and is not influenced by simplifications we made in our simulations. If, for example, oxygen depletion would reduce $q_{s}\;$and hence increase τ res , ELS ¯, a similar decrease in *q*_s_ should naturally occur in a well‐designed SD simulator.

We note that a more comprehensive CFD and metabolic simulation is required for a true quantitative assessment of the process. Still, our approach already yields important insight in the data analysis procedure for Euler–Lagrange simulations, providing lessons regarding the design, operation, and limitations of practical SD simulators. With the presented analysis technique, we acquire insight in the frequency distributions of substrate concentration variations that an SD simulator should replicate, and the challenges associated with this replication. Due to the general nature of a CFD approach, we are confident that these limitations also hold for other organisms, and when including more complex hydrodynamics. With this, we pose a challenge upon the SD community to design a SD simulator that can truthfully mimic variations in large scale bioreactors, at the right magnitude and duration. This can be achieved by running at/above industrial $C_{x}\;$or otherwise by decoupling $\frac{dq_{s}}{dt}$ from *q*_s_ itself. This may (for example) be done by constructing a deliberately nonideal system, by adding an additional dilution stream (with cell retention at the outlet), or by enabling counter‐current exchange between a glucose‐rich and poor plug flow reactor.

## 4 Concluding remarks

We outlined a novel approach to analyse the data acquired by Euler–Lagrange CFD simulations of bioreactors. The Euler–Lagrange approach offers the possibility to analyze substrate concentration variations from the microbial point of view. The obtained $q_{s}\left(t\right)$ series, referred to as lifelines, are divided into metabolic regimes which represent a certain consistent response in an organism's metabolism to the extracellular substrate concentration. By recording the residence time distribution within each regime, how microorganisms switch between regimes, and the duration and magnitude of uptake variations within these regimes, we provide a comprehensive statistical assessment of the substrate fluctuations experienced by organisms in an industrial scale fermentation. This information provides a basis for the design of scale‐down simulators: lab‐scale studies aimed at mimicking industrial scale conditions.

We studied the fermentation of *P. chrysogenum* in a 54 m^3^ stirred vessel with simplified hydrodynamics, neglecting aeration and non‐Newtonian rheology, and assuming no oxygen limitations. The calculated circulation time was in good agreement with the experimental value in water and, despite the simplifications, in reasonable agreement with that in aerated broth. Hence, we are confident the duration and amplitude of substrate concentration variations observed in the CFD simulations are of the correct order of magnitude.

Due to the low $K_{s}\;$value for *P. chrysogenum*, a steep substrate concentration gradient was observed, with 57% of the vessel depleted of substrate. Microorganisms alternate between regions with excess substrate and substrate depletion on timescales of seconds to tens of seconds; of the same order of magnitude as the global circulation time. The rate of change in conditions along the trajectory follows the rate of change expected from the substrate consumption rate $q_{s}\;$with the addition of local dilution effects. This dictates that a SD simulator should operate *at least* at the industrial biomass concentration, or that the local rate of change in substrate concentration should be decoupled from *q*_s_ to allow for more rapid variations than allowed by consumption alone.

The next step toward rational design of scale‐down simulators is to use this CFD data as a basis of design. However, the rapid substrate concentration dynamics observed may prove difficult to replicate in typical SD simulators, specifically in the commonly applied multicompartment approach.

**Practical application**

Scale‐down simulators are a popular tool to study microorganisms under industrially representative conditions. However, little is typically known about the conditions microorganisms encounter in industrial fermentors. Consequently, there is no basis for the rational design of scale‐down simulators, and it is doubtful whether such simulators properly represent industrial conditions.

We have developed a CFD approach that allows to computationally study the dynamic environment in large‐scale fermentors, from the organism's point of view. From this perspective, we obtain easy access to the magnitudes and timescales of fluctuations in the fermentation environment, as experienced by the microorganisms.

These statistics provide a basis for the rational design of scale‐down simulators, that truthfully mimic the variations in the environment (for example in nutrient concentration, temperature, pH), as experienced by microorganisms in industrial scale fermentors.

## Nomenclature

**Table:** 

$C_{s}$	[mol/kg]	Substrate concentration (in broth)
$C_{x}$	[*C*mol*_x_*/kg]	Biomass concentration (in broth)
*D*	[m]	Impeller diameter
***D***	[m^2^/s]	Diffusion coefficient
$d_{s}$	[m]	Diameter of stirrer shaft
*F*	[mol/s]	Substrate feed rate
*H*	[m]	Broth height
*k*	[m^2^/s]	Turbulent kinetic energy
$k_{dE}$	[h^−1^]	Enzyme decay rate
$K_{s}$	[mol*_s_*/kg]	Affinity constant for substrate
$K_{p}$	[mol*_s_*/kg]	Substrate repression constant.
$Y_{sx}$	[*C*mol*_x_*/mol*_s_*]	Max. biomass yield on substrate
$Y_{sp}$	[mol*_p_*/mol*_s_*]	Max. product yield on substrate
$m_{s}$	[*C*mol*_s_*/*C*mol*_x_*/hr]	Maintenance coefficient
*M*	[Nm]	Impeller torque
$M_{arc}$	[‐]	Maximum magnitude of fluctuation along an arc
$M_{s}$	[g/mol]	Molar mass of substrate
$N_{c}$	[‐]	Total number grid cells
$N_{p}$	[‐]	Total number particles
$N_{s}$	[s^−1^]	Impeller speed
$q_{p}$	[mol*_p_*/*C*mol*_x_*/hr]	Specific formation rate of product
$q_{s}$	[mol*_s_*/*C*mol*_x_*/s]	Specific uptake rate of substrate
$Q_{g}$	[m^3^/s]	Gas flowrate
$r_{s}$	[mol*_s_*/m^3^/s]	Volumetric reaction rate of substrate
$S_{s}$	[mol*_s_*/kg/s]	Source term of substrate
*T*	[m]	Tank diameter
*t*	[s]	Time (general)
***u***	[m/s]	Velocity vector
$U_{sup,gas}$	[m/s]	Superficial gas velocity
|U|	[m/s]	Velocity magnitude
*V*	[m^3^]	Broth volume
$q_{s,max}$	[mol*_s_*/*C*mol*_x_*/s]	Max. biomass specific uptake rate of substrate
β	[mol/*C*mol*_x_*/hr]	Reaction constant
Δt	[s]	Timestep
ΔC	[m]	Off‐bottom clearance
ε	[m^3^/s]	Turbulent energy dissipation
μ	[h^−1^]	Specific growth rate
$\nu_{l}$	[m^2^/s]	Kinematic molecular viscosity
$\nu_{t}$	[m^2^/s]	Kinematic turbulent viscosity
ρ	[kg/m]	Density
$\tau_{r,s}$	[s]	Uptake timescale of substrate
$\tau_{lg}$	[s]	Lagrangian timescale
$\tau_{circ}$	[s]	Circulation timescale
τ_95_	[s]	Mixing time
$\tau_{arc}$	[s]	Arc‐time
$\tau_{res}$	[s]	Residence time
$\tau_{res,\;A\;}$	[s]	Mean residence time, regime A.
$\tau_{qp}$	[h]	Adaptation time of $q_{p}$
arc	[‐]	Quantity along arc
*c*	[‐]	Gridcells (Eulerian)
eff	[‐]	Effective
id	[‐]	Ideal mixing
*p*	[‐]	Product
*P*	[‐]	Particles (Lagrangian)
res	[‐]	Residual
*s*	[‐]	Substrate
SS	[‐]	Steady state
*t*	[‐]	Turbulent
*T*	[‐]	Tank
		
*x*	[‐]	Biomass
*E*	[‐]	Excess (regime)
*L*	[‐]	Limitation (regime)
*S*	[‐]	Starvation (regime)
$\bar{y}$	[‐]	Volume‐average of *y*
p(y)	[‐]	Probability of *y*
P(y)	[‐]	Probability of *y* (cumulative)
σ(y)	[‐]	Standard deviation of *y*
Po	[‐]	Power number
Re	[‐]	Reynolds number
Sc	[‐]	Schmidt number
$Sc_{t}$	[‐]	Schmidt number, turbulent
St	[‐]	Stokes number

*The authors have declared no conflicts of interest*.

## Supporting information

As a service to our authors and readers, this journal provides supporting information supplied by the authors. Such materials are peer reviewed and may be re‐organized for online delivery, but are not copy‐edited or typeset. Technical support issues arising from supporting information (other than missing files) should be addressed to the authors.

Appendix A: Modified kinetic model by Douma et al.
Table A.1: Parameters of the black box kinetic model applied in this study [1‐3].
Figure B.1: Regime maps for different circulation times, achieved by altering the impeller velocity.
References for the appendices
