# Supplementary material for: Euler‐Lagrange computational fluid dynamics for (bio)reactor scale down: An analysis of organism lifelines
Source: Eng Life Sci. 2016 Sep 14;16(7):652–63. doi: 10.1002/elsc.201600061 (PMC5129516; doi:10.1002/elsc.201600061)
Supplement: Supplementary file 1 — Appendix A: Modified kinetic model by Douma et al. Table A.1: Parameters of the black box kinetic model applied in this study [1‐3]. Figure B.1: Regime maps for different circulation times, achieved by altering the impeller velocity. References for the appendices [file ELSC-16-652-s001.pdf]

**Supporting information A: Modified kinetic model by Douma et al.**

Douma et al. [1] based several kinetic parameters for the model equations (section 2.1) on work by van Gulik et al. [2]. More recently, De Jonge et al. re-evaluated the kinetic parameters, with improved methods for determining the residual $C_s$ [3], yielding $q_{s,max}$ and $K_s$ as reported in table S.1. For consistency, this means $K_p$ in eq. 2 must be refitted, yielding $K_p = 8.36 \cdot 10^{-6}$ $mol_s$/kg The updated model parameters are shown in table S.1. In eq. 2, $q_p$ is proportional to the availability of limiting enzyme; the first term represents enzyme synthesis, the second represents decay and dilution. In the Herbert-Pirt equation (eq. 3) $\mu$ is negative if $q_s < q_p/Y_{sp} + m_s$ which can be interpreted as consumption of stored carbon, or cell death due to starvation; fundamentally, a negative $\mu$ is not problematic. However, $\mu < 0$ is a problem in eq. 2. Synthesis becomes negative for $\mu < 0$, which is not deemed realistic and can lead to a negative $q_p$ giving numerical instabilities. In the second term of eq. 2, $\mu < 0$ gives rise to an unrealistic enzyme increase. We propose two simple modifications to prevent this behaviour. The first term is set to 0 for $\mu < 0$ to prevent negative synthesis. In the second term, $\mu$ is replaced by $|\mu|$ such that $\mu < 0$ leads to enzyme destruction rather than concentration. We stress there is no experimental backing for these choices, but we deem them defendable for the goal of our investigation. The modified version of eq. 2 is eq. S.1:

$$\frac{dq_p}{dt} = \frac{\beta \cdot \max(0,\mu)}{1 + \left(\frac{C_S}{K_p}\right)^m} - (K_{dE} + |\mu|)q_p \qquad \text{(S.1)}$$

Table S.1: Parameters of the black box kinetic model applied in this study [1-3].

| **Parameter** | **Value** | **Unit** |
|---|---|---|
| $q_{s,max}$ | $12.47 \cdot 10^{-6}$ | $mol_s$/C$mol_x$/s |
| $K_s$ | $7.8 \cdot 10^{-6}$ | $mol_s$/kg |
| $K_p$ | $8.36 \cdot 10^{-6}$ | $mol_s$/kg |
| $k_{dE}$ | 0.0147 | $hr^{-1}$ |
| $\beta$ | $8 \cdot 10^{-4}$ | $mol_p$/C$mol_x$/s |
| $Y_{sx}^{max}$ | 3.96 | $mol_x$/$mol_s$ |
| $Y_{sp}^{max}$ | 0.174 | $mol_p$/$mol_s$ |
| $m_s$ | 0.0015 | $mol_s$/C$mol_x$/s |
| $m_x$ | 28.05 | g/$mol_x$ |

**Influence of fluctuations in the starvation regime on $q_p$:** It was assumed that the absolute value of $q_p$ is irrelevant in the starvation regime, where $q_s/q_{s,max} < 0.05$. No saturation of $q_s$ occurs in the starvation regime; $q_s$ is a linear function of $C_s$ and order-of-magnitude changes in $q_s$ will occur for $q_s/q_{s,max} < 0.05$. The assumption that fluctuations in $q_s$ are irrelevant in the starvation domain, is based on the small absolute value of $q_s$ here. Calculating $q_p$ using the approach of section 3.3, but replacing all values of $q_s/q_{s,max} < 0.05$ with $q_s = 0$ yields $q_p = 0.77 \cdot 10^{-4}$, equal to $q_p$ reported in section 3.3. Setting all values $q_s/q_{s,max} < 0.05$ equal to $0.05$ then the result is $q_p = 0.89 \cdot 10^{-4}$, a small change compared to the value observed before, within an acceptable margin of error. These results show that fluctuations inside the starvation regime will have a small effect on the calculated $q_p$ and that they indeed can be neglected.

**Supporting information B: Fluid dynamic setup and validation**

*Geometry*

The main geometric details were reported in section 2.2. Parameters not mentioned in the main article include the shaft diameter $d_s = 0.267$ m, the shaft is extended through the entire tank. The stirrer off-bottom clearance $C = 0.9$ m and mutual impeller clearance $\Delta C = 3$ m. Four baffles (width $T/10$) were placed over the entire vessel height. A gas sparger (circular) with an outer diameter of $D$ and inner diameter of $2D/3$ was placed at an off-bottom clearance $C/2$. A cooling coil in the original geometry was omitted for simplicity. The feed point was mimicked by defining substrate source terms in a $0.034$ m$^3$ box, centred at [$r$ (m), $Y$ (m), $\theta$ (degrees)] = $[0.80, 7.35, 165]$, the angle with respect to the positive $x$ direction. This is a simplification as the real feed tube is too small to resolve. Preliminary work showed that the size of the feed region, provided it is much smaller than the tank volume, has little effect on the shape and size of the metabolic regime distribution due to rapid initial dilution. The baffles and impellers were modelled as 0-thickness walls [4]. All walls have a no-slip boundary condition, the top was a no-shear surface. The shaft was a moving (no slip) wall with an angular velocity equal to the impeller rotation speed.

*Fluid dynamics model*

Based on the favourable results of Gunyol and Mudde [4] and Coroneo et al. [5] the standard $k - \varepsilon$ was chosen, yielding reasonable predictions for $k, \varepsilon$ and the mixing time. For details, [4] can be consulted. The simple gradient hypothesis is used to link the turbulent diffusion of species ($D_t$) to $\nu_t$; via the turbulent Schmidt number $Sc_t = \nu_t / D_t$. As observed by, among others, Montante et al. and Gunyol et al. [6, 7], setting $Sc_t = 0.2$ provides a better agreement with experimental data. Our observations are in agreement with these findings. The local reaction rate is based on the mean substrate concentration inside a gridcell, $\overline{C_{s,c}}$. This ignores possible sub-grid concentration gradients; preliminary work at higher mesh densities showed sub-grid gradients have a negligible effect on the overall results. The organism size is approximately $d_p = 5 \cdot 10^{-6}$ m [8], giving a Stokes number $St \equiv \frac{\rho_p d_p u_p}{18 \nu_l \rho_l} < 0.01$; hence, particles can be treated as ideal flow followers [9]. Particle-turbulence was invoked via the *Discrete random walk* (DRW) model, where a random velocity is superimposed on the mean velocity, determined from:

$$\tau = \min\left(-C_L \frac{k}{\varepsilon} \log(r), \quad -\tau_r \ln\left[1 - \frac{L_e}{\tau_r |u_f - u_p|}\right]\right) \tag{S.2}$$

$$u' = \chi \sqrt{\frac{2k}{3}} \tag{S.3}$$

Here, $\tau_r$ is the particle relaxation time, $L_e$ the eddy length-scale and $r$ a random number between 0 and 1. The constant $C_L$ is linked to $Sc_t$, and is set to 0.45 rather than the default 0.15 to reflect the change in $Sc_t$. $\chi$ is a normally distributed random number. Turbulence is assumed to be isotropic. A known issue with the DRW is that near walls $k \rightarrow 0$, resulting in particles artificially 'sticking' to walls. This can be solved by using an alternative calculation for $D_t$ [10] or using an improved random walk model [11]. In our simulations, sticking was of minor concern as is reflected in the regime distributions (table 2) and improvements over the $DRW$ were deemed unnecessary. $2^{nd}$ order upwind discretization was used for all equations [4]. The SIMPLE algorithm was used for pressure-velocity coupling. For transient simulations, second order implicit discretization in time was applied. For particle tracking, a trapezoidal tracking scheme with an automatically adapting particle timestep $t_p < \Delta\, t_s$ was applied. Subroutines to store the observed substrate concentration and track the development of $\mu$ and $q_p$ per particle were added via user defined functions (UDFs).

**Mass transfer limitations:** The results of Linkes et al. [12] indicate that sub-grid concentration variations due to turbulence lead to only small fluctuations in $q_s$, even for cells in the vicinity of $K_s$. As such, basing the reaction rate on $\overline{C_{s,c}}$ leads to no significant error with respect to turbulent concentration variations either (definitely not compared to the inherent approximations and limitations of turbulence modeling). For simplicity, we currently omitted mass transfer limitations from the bulk to the organism, which may in practice lead to lower local concentrations than the local bulk concentration. If desired, such effects can be straightforwardly included in the reaction term via common closure relations.

*Solution strategy and convergence criteria*

First, the hydrodynamics were solved; convergence was declared when $\overline{|U|}$ was stable within $0.01\%$ and the residuals were below $10^{-5}$. Next the flowfield was frozen and the mixing time was determined in a transient simulation with a timestep size $\Delta t = 20$ ms initially patching the feed zone with tracer. We monitored the mixing time with a probe near the bottom, at coordinates [$r$ (m), $Y$ (m), $\theta$ (degrees)] = $[0.7\,, 0.25, 45]$. The concentration gradient with reaction was determined in steady-state. When converged $\overline{C_s}$ was stable within $0.01\%$ over $500$ subsequent iterations. The gradient was then frozen and particles were added to the domain and tracked in a transient simulation with $\Delta t = 15$ ms. First the particles are mixed for $70$ s ($> \tau_{mix}$) to ensure a homogeneous distribution. Subsequently the tracking scripts were added. The

sampling timestep was set to $\Delta t = 30$ ms to ensure $\Delta t < \tau_{r,lim}/10$, providing reasonable resolution even in the most extreme cases.

*Validation*

**Turbulence:** The simulated impeller power numbers $Po = \frac{2\pi N_s M}{\rho N_s^3 D^5}$ are 5.19 and 6.28 for top and bottom respectively, using the crudest mesh of 235000 gridcells. Both are well in line with measurements by Wu and Pullum, who report values of 5.6 and 6.6 for 6 and 8 blades, respectively [13]. The observed $k$ and $\varepsilon$ profiles in the top impeller discharge stream were in excellent agreement with Gunyol and Mudde [4] and hence the experiments of Wu and Patterson [14]; the profiles are not shown for brevity. It must be noted that Wu and Patterson computed $\varepsilon$ as $\varepsilon = 0.85(k^{\frac{3}{2}})/L_r$. Direct measurements of $\varepsilon$ by Ducci et al. show stronger dissipation near the impeller tip [15], but decent agreement in the bulk. It is established that the $k - \varepsilon$ model cannot properly capture the flow close to the impeller [16]. As the impeller region is relatively small, this will have limited effect on the overall mixing. The $k - \varepsilon$ model is strongly preferred over more elaborate turbulence models, to keep the calculation time tractable.

**Mixing time:** The experimental $\tau_{circ}$ is compared with the simulated $\tau_{circ}$ in table B.1. Experimentally, the lag time between injection (top) and detection (bottom) was measured, which is equal to $\tau_{circ}/2$. In FLUENT the lag time was determined as the time before $C_s/\overline{C_s} > 0.01$ was detected at the bottom. The $\tau_{circ}$ is found to be mesh-independent and in reasonable agreement with the experimental value of 9.63 s. The mixing time, $\tau_{95}$ shows good agreement between the meshes, too. The results are in reasonable agreement with the rule of thumb $\tau_{95} \approx 4\tau_{circ}$ [17]. Experimental data for $\tau_{95}$ was, however, unavailable.

**Mesh dependency:** In table S.2 it is clear there is no influence of the mesh resolution on mixing time (a mesh of 803000 cells yielded $\tau_{95} = 63.5$). $Po$ for the 401k mesh was 5.35 and 6.42 for top and bottom, respectively, and the regime layout was very similar in all meshes. As such, we deem the crudest mesh of 235000 gridcells sufficiently accurate for our simulations.

Table S.2: A comparison of mixing times between the meshes. $t_1$ shows the onset of mixing ($C_{s,c}/\overline{C_s} > 0.01$), $t_{95}$ the 95% mixing time based on a single monitor point probe.

| No. gridcells | $t_1$ [s] | $t_{95}$ [s] |
|---|---|---|
| 235000 | 18.2 | 63.8 |
| 401000 | 18.2 | 63.4 |
| experimental | 19.3 | |

*Regime map sensitivity*

In our work we simulated the fluid as single phase water, rather than the true aerated fermentation broth. As a consequence the simulated circulation time is somewhat shorter than the true circulation time. To provide some insight in how a longer circulation time may change the regime layout, the simulation was also conducted at stirring speeds of 1.17 $s^{-1}$ and 0.7 $s^{-}$, further referred to as case '*N1.17*' and '*N0.7*'. These yield circulation times of 25.6 s and 42.8 s, in line with the measured value for aerated water and broth, respectively. The regime map change is shown in figure S.1. For case *N1.17* the change is minor, a slight increase of the excess regime due to slower mixing close to the feedpoint, and a slight increase of the starvation zone, shifting the starvation-limitation boundary slightly upward. For *N0.7*, the excess zone is significantly more pronounced, and the limitation-starvation boundary shifted further upwards.

The change in residence time distributions was studied by tracking 4300 particles over 3600 seconds for both cases. The lower number of particles was chosen to limit runtime, making the statistics more noisy, but sufficiently clear to distil the trends. To facilitate comparison with the original data, all distributions are normalized. The most significant changes occur in the excess zone, figure S.2 A, where the increase in size of the zone and lower particle velocity leads to a similarly shaped but broader time distribution. Due to the superposition of increased size and lower velocity, the stretching is not proportional to the change in stirring rate ($\tau_{LEL,18.2} = 3.65$ s $\tau_{LEL,N1.17} = 7.05$, $\tau_{LEL,\mathrm{N0.7}} = 12.45$ s). In the limitation zone, all distributions were still similarly shaped for longer times, hence only the $ELS$-type is shown for brevity (at short times, there were some changes due changes around the top impeller). The distribution becomes slightly wider for case *N1.17*, but then remains relatively unchanged for *N0.7*. To explain this, we refer back to section 3.7 of the paper, where we argued that from the microbial point of view, the rate of change in $q_S$ followed from consumption plus local mixing. The higher $\tau_{circ}$, the lower the contribution of mixing, and the closer to ideal plug flow the system operates. Consequently, the mean residence time for $ELS$ approaches the time required to move from

$q_s/q_{s,max} = 0.95$ to $q_s/q_{s,max} = 0.05$ in the limit $\tau_{circ} \to \infty$, which is 8.13 s in this case. Indeed, the mean $ELS$-residence time was found to be $\tau_{ELS,N1.17} \approx 8.3$ s and $\tau_{ELS,N0.7} \approx 8.0$ s which are in agreement with this reasoning.

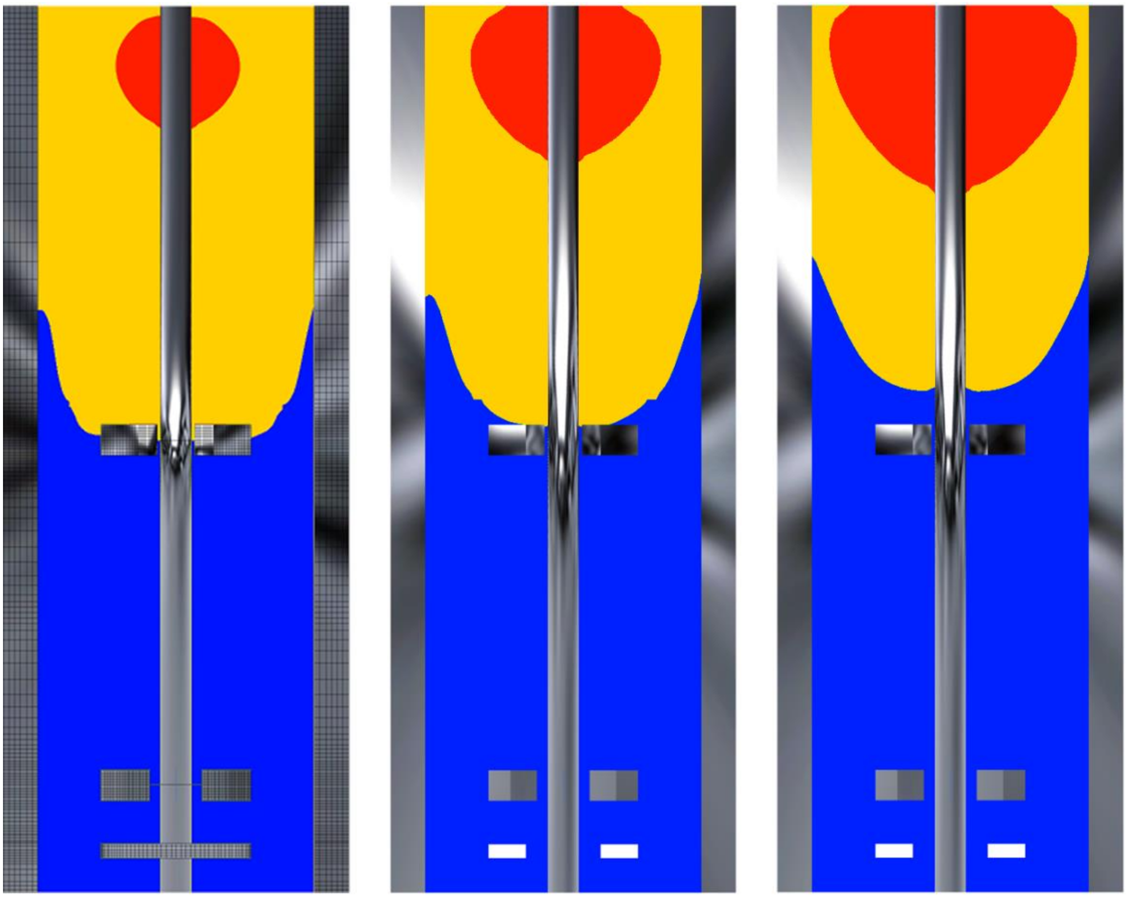

Figure S.1: Regime maps for different circulation times, achieved by altering the impeller velocity. Left: regular settings, $\tau_{circ} = 18.2$ s. Middle: *N1.17* ( $\tau_{circ} = 25.6$ s). Right:  Middle: *N0.7* ( $\tau_{circ} = 42.8$ s).

In the starvation zone, not only broadening of the distribution occurs, but the changes close to the top impeller lead to a slightly different shape altogether. For *N1.17* the peak at 1 second is reduced, and slightly longer trajectories are more common because the impeller disc and regime boundary no longer overlap. The lower broth velocity changes the slope of the long-time distribution, although the limited number of counts lead to significant noise in this region. The weaker slope for the long-term distribution is even more pronounced for *N0.7*, where the short-term peak is nearly completely removed due to the top impeller being fully in starvation.

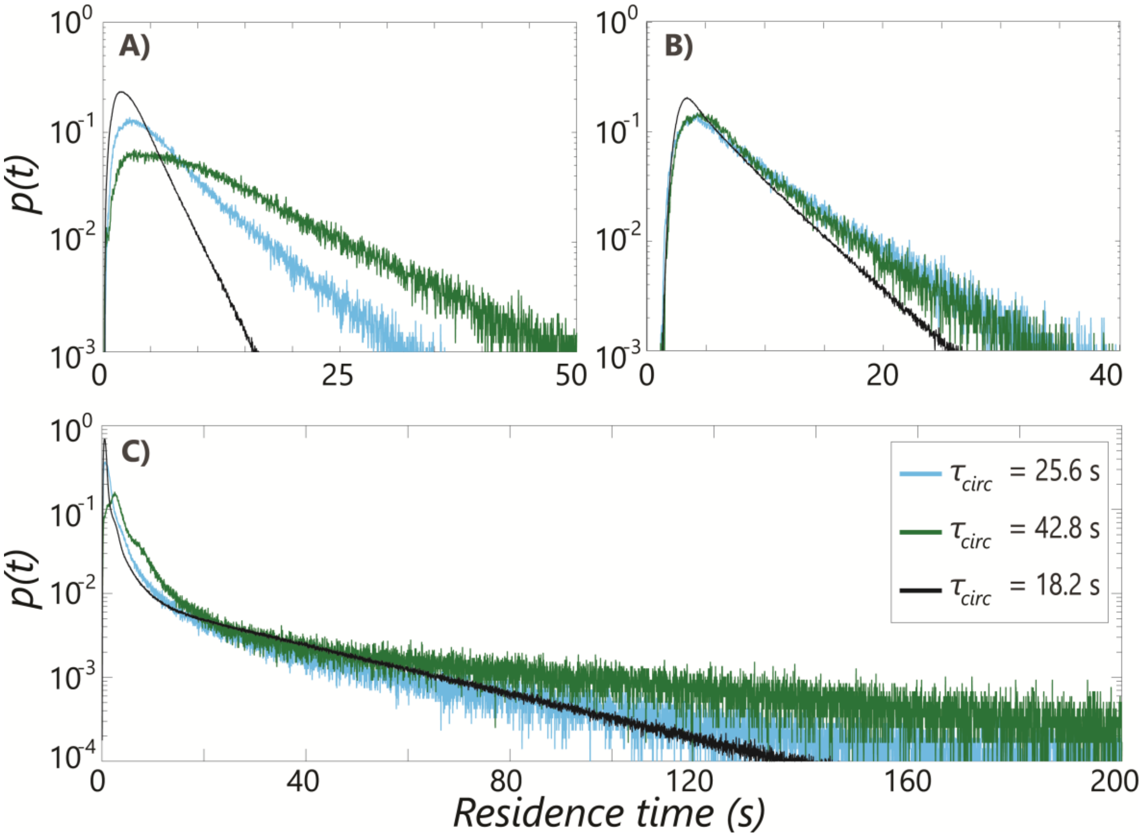


Figure S.2: Sensitivity of the regime residence time distributions to the circulation time. **A)** *LEL*-transition; the increasing size of the excess regime strongly affects residence time. **B)** *ELS*-transition; a small effect is visible due to changes in local mixing. **C)** *LSL*-transition; changes in the distribution mainly occur due to the immersion of the top impeller in the starvation regime

With this additional data, we show that the circulation time indeed has some impact on the per-regime residence time distribution, leading to stretching of the distribution if the flow pattern inside the regime is not altered, while changing the distribution shape when the flow pattern inside the regime is altered, in this case by inclusion of a circulation loop. Second, the results discussed here do not include the possible changes in flow pattern due to aeration and rheology, which may lead to different circulation behaviour even at the same global circulation time. Also, reducing the agitation rate reduces power input which influences particle movement. As such, the comparison shown here is to illustrate possible changes only. The dominant conclusions, however, remain unaltered: concentration variations occur in the order of magnitude of the circulation time, and to properly assess the rate-of-change in conditions, ideal simulators should operate at industrial biomass concentrations.

## Supporting information C: Treatment of turbulent motions

*Filtering of turbulent fluctuations*

The turbulent movement of particles leads to rapid variations in $q_s/q_{s,max}$, with rates of change in $q_s$ an order of magnitude faster than can be replicated in an ideal. These rapid $q_s/q_{s,max}$ variations will introduce features in the regime residence time distributions and limitation regime dynamics, that cannot be addressed feasibly with conventional scale down simulators. Hence, these turbulent variations must be filtered to attain tractable distributions. We distinguish three types of rapid $q_s$ variations: rapid successive regime crossings, integral scale $q_s$ variations and subgrid $q_s$ variations by small eddies. Based on the work of Linkes et al. [12] the latter was neglected. The other two effects both result from the DRW model, occurring at the Lagrangian timescale $\tau_{lg} = 0.45k/\varepsilon$. Timescale $\tau_{lg}$ locally varies from 0.007-1.5 s with $\overline{\tau_{lg}} = 0.36$ s. Comparison with the reaction timescale $\tau_{r,s} = \frac{C_s}{C_x q_s}$,which gives 0.32 s for $C_s \to 0$, hints that turbulent variations in $q_s$ may be significant *within* integral-scale eddies. Whether or not the metabolism responds significantly to such rapid, high magnitude variations is an interesting topic for further study, but not considered here.

For a tractable analysis, the turbulent variations need to be separated from the global trajectory and treated as superimposed variations. This is done by constructing a smoothed $q_s$ −timeseries, using a moving-average filter, with a filter time $\overline{\tau_{lg}} = 0.36$ s. All figures in the main text of this document are based on the smoothed data. Second we consider rapid successive regime transitions. This issue is separate from the turbulence issue outlined above - the smoothed data may still exhibit rapid consecutive regime crossings. Especially with steady-state extra-cellular conditions as applied here, the turbulent motions cause particles to frequently hop back-and-forth over the fixed regime boundaries (in reality, the background field is also dynamic, of course). In rapid consecutive transitions, particles will not venture far from the regime boundary and the variations will have a low amplitude in $q_s$. Hence, to remove them we set a 'fuzzy regime boundary' filter with an amplitude of $\pm$ 0.01 in $q_s/q_{s,max}$. For example, consider the starvation regime boundary (at $\frac{q_s}{q_{s,max}} = 0.05$). For a transition from starvation to limitation to count, a particle must cross the boundary $\frac{q_s}{q_{s,max}} = 0.06$, and for limitation to starvation, $\frac{q_s}{q_{s,max}} = 0.04$ must be crossed. Hence, the regime boundary filter acts to remove low-amplitude variations around regime boundaries. This is visually represented in figure S.3. The effect of filtering on the overall distribution is included in table 2; with both filters applied the deviation from raw data is less than 5% for all regimes, which we deem acceptable.

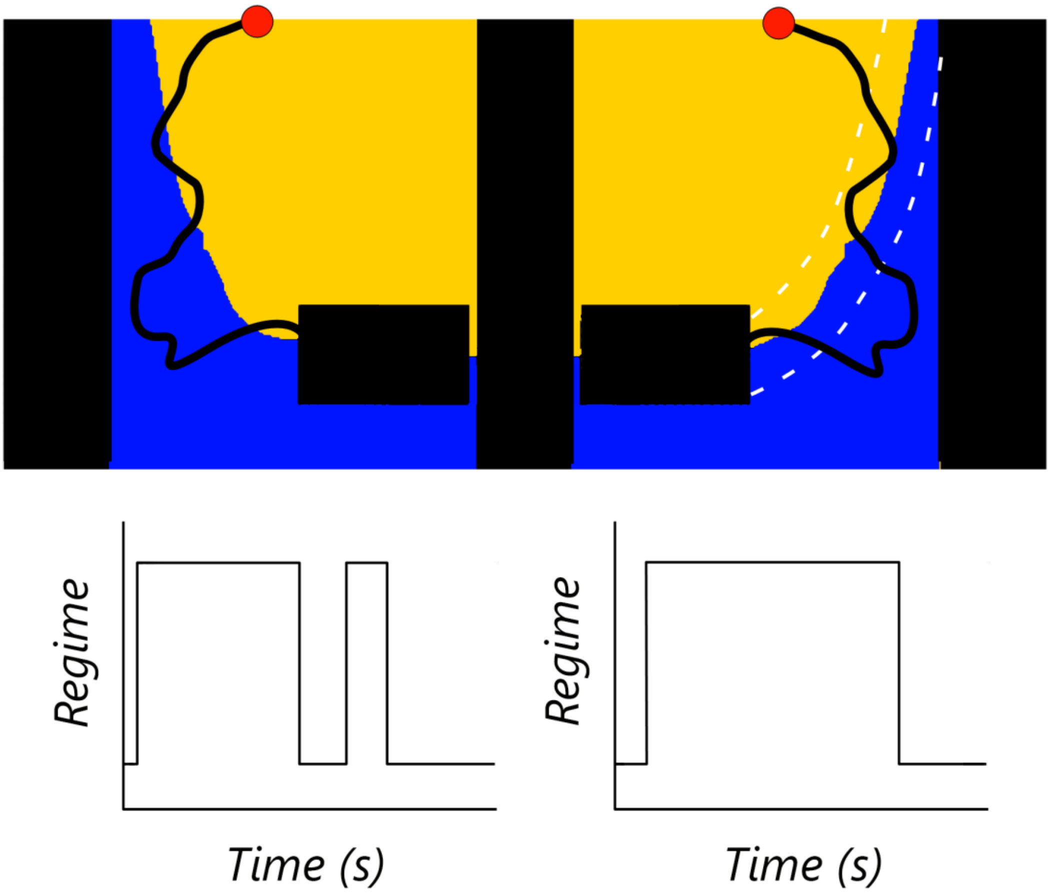


Figure S.3: Graphical representation of fuzzy-boundary filtering. **A)** The track of a particle translated into a regime-plot, unfiltered. **B)** The same track with turbulence filtering. Turbulence filtering is performed solely on the magnitude of the observed $q_s$, not on the duration of a fluctuation.

*Statistics of turbulent $q_s$ fluctuations*

In the main text, we worked under the assumption that rapid fluctuations in $q_s$ caused by turbulence would not significantly affect the metabolism; in any case, they'll be difficult to reproduce in scale down simulators. Hence, we did not address an analysis of turbulent movements in the main text. For the interested reader, we here present briefly a methodology to assess the statistics of turbulent movements. The magnitude of turbulent movements can be determined by subtracting the smoothed $q_s$ signal (called $q_{s,smooth}$) from the raw signal. The data displayed in figure S.4 is based on the analysis of 25000 particles. We determine the probability distribution of the turbulent movements, $q_{s,t}$ around the mean $q_s$ that follows from the smoothed data (figure S.4 A) which is narrow near the boundaries of $q_{s,smooth}$ and has a maximum width at $q_{s,smooth} = 0.5q_{s,max}$. This behavior is reflected in the distribution standard deviation $\sigma$ and is consistent with the saturation of kinetics at both extremes in $q_s$. The

skewness is close to 0 at low $q_{s,smooth}$, and increases with $q_s$ due to the shift from $1^{st}$ to $0^{th}$ order kinetics. The increase in asymmetry with increasing $q_{s,smooth}$ is also visible when fitting the turbulent $q_s$ distribution with a symmetric trial function; the symmetric Gaussian- and Laplace-distribution are used. Over the entire range, the sharper peak of the Laplace distribution provides a better approximation of the observed data (figure S.4 E) which is in accordance with motion on an exponentially distributed random time (which applies for the *DRW*) following this distribution. To account for the effect of the shifting kinetics, an asymmetric Laplace distribution may give a better fit.

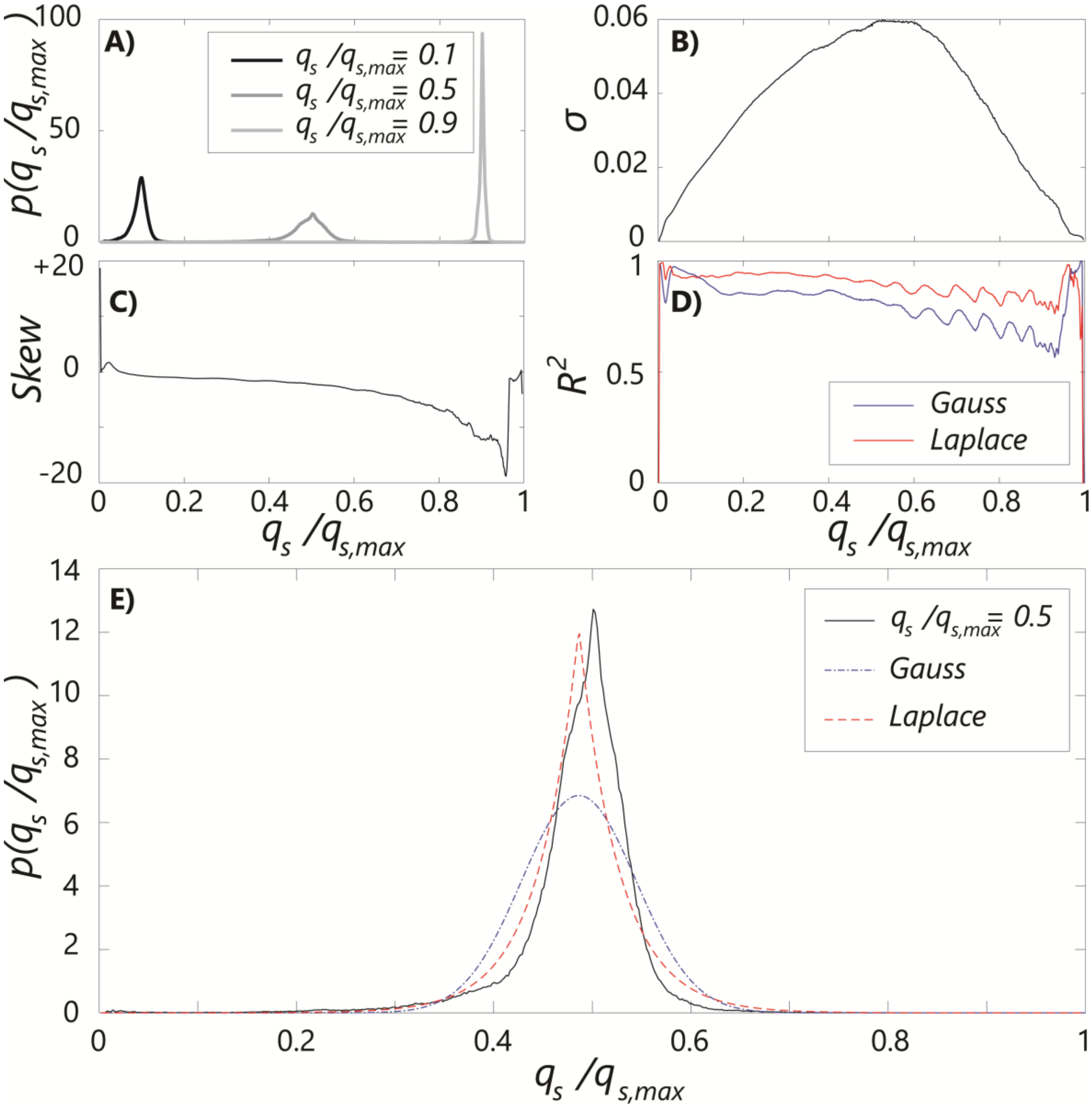


Figure S.4 **A)** Turbulent $q_s$ distribution at three values of smoothed $q_s$. **B)** Standard deviation of the turbulent $q_s$ distribution as a function of smoothed $q_s$. **C)** Skewness of the turbulent $q_s$ distribution as a function of smoothed $q_s$. **D)** $R^2$ for the fit of a Gaussian (red) and Laplacian distribution to the turbulent $q_s$ distribution. **E)** Comparison of the computed distribution at $q_s/q_{s,max} = 0.5$ with the fitted Gaussian and Laplacian.

**References for the appendices**

[1] Douma, R. D., Verheijen, P. J. T., de Laat, W. T. A. M., Heijnen, J. J. and van Gulik, W. M., Dynamic gene expression regulation model for growth and penicillin production in Penicillium chrysogenum., *Biotechnology and bioengineering*, 2010, *106*, 608–18, doi: 10.1002/bit.22689.

[2] van Gulik, W. M., de Laat, W. T. A. M., Vinke, J. L. and Heijnen, J. J., Application of metabolic flux analysis for the identification of metabolic bottlenecks in the biosynthesis of penicillin-G, *Biotechnology and Bioengineering*, 2000, *68*, 602–618.

[3] de Jonge, L. P., Buijs, N. A. A., ten Pierick, A., Deshmukh, A. et al., Scale-down of penicillin production in Penicillium chrysogenum., *Biotechnology journal*, 2011, *6*, 944–58, doi: 10.1002/biot.201000409.

[4] Gunyol, O. and Mudde, R. F., Computational Study of Hydrodynamics of a Standard Stirred Tank Reactor and a Large-Scale Multi-Impeller Fermenter, *International Journal for Multiscale Computational Engineering*, 2009, *7*, 559–576.

[5] Coroneo, M., Montante, G., Paglianti, A. and Magelli, F., CFD prediction of fluid flow and mixing in stirred tanks: Numerical issues about the RANS simulations, *Computers & Chemical Engineering*, 2011, *35*, 1959–1968, doi: 10.1016/j.compchemeng.2010.12.007.

[6] Montante, G., Moštek, M., Jahoda, M. and Magelli, F., CFD simulations and experimental validation of homogenisation curves and mixing time in stirred Newtonian and pseudoplastic liquids, *Chemical Engineering Science*, 2005, *60*, 2427–2437, doi: 10.1016/j.ces.2004.11.020.

[7] Gunyol, O., Noorman, H. J. and Mudde, R. F., CFD simulations of a large-scale fermenter with muliple impellers, in: Chaouki, J., Tanguy, P. (ed.), *Proceedings of the 9th International Conference on Gas-Liquid Solid Reactor Engineering*, Montreal, 2009 pp. 1–4.

[8] Deshmukh, A. T., *Elucidation and modeling of the in-vivo kinetics of enzymes and membrane transporters associated with beta-lactam and non-ribosomal peptide production in P. Chrysogenum*, Phd thesis, Delft university of Technology, 2013.

[9] Lapin, A., Müller, D. and Reuss, M., Dynamic Behavior of Microbial Populations in Stirred Bioreactors Simulated with EulerLagrange Methods: Traveling

along the Lifelines of Single Cells, *Industrial & Engineering Chemistry Research*, 2004, *43*, 4647–4656, doi: 10.1021/ie030786k.

[10] Lapin, A., Schmid, J. and Reuss, M., Modeling the dynamics of E. coli populations in the three-dimensional turbulent field of a stirred-tank bioreactor: A structured segregated approach, *Chemical Engineering Science*, 2006, *61*, 4783–4797, doi: 10.1016/j.ces.2006.03.003.

[11] Dehbi, A., A CFD model for particle dispersion in turbulent boundary layer flows, *Nuclear Engineering and Design*, 2008, *238*, 707–715, doi: 10.1016/j.nucengdes.2007.02.055.

[12] Linkès, M., Fede, P., Morchain, J. and Schmitz, P., Numerical investigation of subgrid mixing effects on the calculation of biological reaction rates, *Chemical Engineering Science*, 2014, *116*, 473–485, doi: 10.1016/j.ces.2014.05.005.

[13] Wu, J., Zhu, Y. and Pullum, L., Impeller Geometry Effect on Velocity and Solids Suspension, *Chemical Engineering Research and Design*, 2001, *79*, 989–997, doi: 10.1205/02638760152721857.

[14] Wu, H. and Patterson, G., Laser-Doppler measurements of turbulent-flow parameters in a stirred mixer, *Chemical Engineering Science*, 2989, *44*, 2207–2221, doi: 10.1016/0009-2509(89)85155-3.

[15] Ducci, A. and Yianneskis, M., Direct determination of energy dissipation in stirred vessels with two-point LDA, *AIChE Journal*, 2005, *51*, 2133–2149, doi: 10.1002/aic.10468.

[16] Singh, H., Fletcher, D. F. and Nijdam, J. J., An assessment of different turbulence models for predicting flow in a baffled tank stirred with a Rushton turbine, *Chemical Engineering Science*, 2011, *66*, 5976–5988, doi: 10.1016/j.ces.2011.08.018.

[17] Noorman, H., An industrial perspective on bioreactor scale-down: what we can learn from combined large-scale bioprocess and model fluid studies., *Biotechnology journal*, 2011, *6*, 934–43, doi: 10.1002/biot.201000406.
